# Refractures in Children

**DOI:** 10.2106/JBJS.24.01014

**Published:** 2025-03-27

**Authors:** Oskari Pakarinen, Matti Ahonen, Petra Grahn, Ilkka Helenius, Topi Laaksonen

**Affiliations:** 1 Department of Surgery, Päijät-Häme Central Hospital, Lahti, Finland; 2 Faculty of Medicine, University of Helsinki, Helsinki, Finland; 3 Department of Paediatric Orthopaedics, Helsinki New Children’s Hospital, Helsinki, Finland; 4 Department of Orthopaedics and Traumatology, Helsinki University Hospital, University of Helsinki, Helsinki, Finland

## Abstract

**Background:**

Fractures are common in children, but knowledge about refractures has been limited. This study aimed to determine the rate of radiographically confirmed refractures within 2 years of the primary fracture in children and to analyze the association between fracture stability and refracture risk.

**Methods:**

All patients who were <16 years of age and had at least 2 fractures in the same bone between 2014 and 2023 were reviewed from the Helsinki University Hospitals’ electronic pediatric treatment register, KIDS Fracture Tool. Patients’ radiographs and records were evaluated. Patients with subsequent fractures in different parts of the bone than the primary fracture, patients with pathological fractures, and patients with a systemic condition predisposing to fractures were excluded.

**Results:**

Of 20,749 fractures, 163 consecutive fractures in the same bone within 2 years were identified. After exclusions, 100 cases (0.48% of all fractures) remained, with 83 occurring within 1 year and 17 occurring in the second year after the primary fracture. Refracture rates were highest in diaphyseal both-bone forearm fractures (3.76% [43 of 1,144]), diaphyseal tibial fractures (1.01% [7 of 693]), distal forearm fractures (0.55% [27 of 4,949]), and distal humeral fractures (0.49% [11 of 2,227]). The median time to refracture was 73 days (interquartile range [IQR], 56 to 131 days) for the distal forearm, 109 days (IQR, 79 to 169 days) for the diaphyseal tibia, 124 days (IQR, 80 to 178 days) for the diaphyseal forearm, and 426 days (IQR, 243 to 660 days) for the distal humerus. Displaced fractures requiring closed reduction had a significantly higher refracture risk compared with other fractures: relative risk (RR), 8.0 (95% confidence interval [CI], 4.5 to 14) compared with stable fractures; RR, 5.0 (95% CI, 2.9 to 8.7) compared with fractures that had acceptable position but might be unstable and required follow-up; and RR, 3.2 (95% CI, 1.8 to 5.7) compared with fractures requiring fixation and follow-up.

**Conclusions:**

The overall refracture rate in children was approximately 0.5%, with the highest rates in both-bone diaphyseal forearm fractures. The median time to refracture varied significantly by anatomic location, and displaced fractures treated with closed reduction were associated with a higher refracture risk.

**Level of Evidence:**

Therapeutic Level III. See Instructions for Authors for a complete description of levels of evidence.

Fractures are common in children, with an estimated incidence of 163 to 202 per 10,000 person-years^1-4^. Fractures occur 1.5 to 1.9 times more often in boys than in girls, and upper-extremity fractures represent 73% to 82% of all fractures in children^1-3^. Most pediatric fractures are treated with cast immobilization, either with or without closed reduction. However, the incidence of surgically treated fractures is increasing, particularly in the upper extremity^5^. At the end of the immobilization period, the fractured bone often has not regained full strength because the ossification process is still ongoing, making it more susceptible to refracture. A refracture means a subsequent fracture in the previous fracture site; however, no unambiguous definition exists in the literature. In the *Merriam-Webster Medical Dictionary*^6^*,* a refracture is defined as a “break along the line of a previous fracture.” Cassebaum and Hamilton^7^ defined refracture in 1953 “by the trauma being identical; that the bones had an anatomic site of weakness; or that there remained a point or points of weakness after healing.”

Refractures in children have primarily been studied through small-cohort studies or case series, often focusing on specific anatomic regions, most commonly the forearm, with a reported refracture risk of up to 5%^8-13^. A study from the Swedish Fracture Register reported a 0.88% 1-year incidence of long-bone refractures in children, identifying 348 refractures within the same region as the primary fracture^14^. However, that study could not confirm whether these were true refractures or assess injury mechanisms, treatment methods, or patient-related factors, leaving the understanding of refractures incomplete.

The main aim of this study was to investigate the risk of radiographically confirmed refractures within 1 and 2 years after the primary fracture in children <16 years of age, based on data from the KIDS Fracture Tool (New Children’s Hospital, Helsinki, Finland, and BCB Medical, Turku, Finland) and radiographic analysis. The secondary aim of this study was to analyze how the treatment type of the primary fracture was associated with the risk of refracture.

## Materials and Methods

### Study Setting

New Children’s Hospital is the only tertiary-level hospital in the Helsinki area and the only hospital providing on-call pediatric orthopaedic treatment in Finland. The hospital hosts an electronic pediatric fracture treatment, quality, and register device, the KIDS Fracture Tool, which includes >22,000 fractures since 2014, and it is also linked to the patient records and radiographs. The KIDS Fracture Tool includes data since 2014 on all fractures in children ≤15 years of age, covering birth date, sex, fracture date, injury mechanism, fracture location, treatment, complications, and number of follow-up visits. Additionally, the registry roughly categorizes fractures by severity into 4 groups to guide treatment and follow-up:

- Group A: Acceptable fracture position that does not require follow-up.
- Group B: Acceptable fracture position that may be unstable and requires follow-up.
- Group C: Displaced fracture requiring closed reduction and follow-up.
- Group D: Displaced fracture requiring fixation and follow-up.

Fracture energy increases from Group A to Group D. Group A mainly includes torus fractures, whereas Group D includes high-energy injuries, displaced intra-articular fractures, and cases in which closed reduction has failed or fixation is needed because of the patient’s bone maturity. Examples of fractures in each group can be found in Appendices 1 and 2.

Although the completeness of the KIDS Fracture Tool has not been formally validated, private clinics in the region lack on-call anesthesia and pediatric orthopaedic services. As a result, children with displaced fractures are referred to the New Children’s Hospital, ensuring that the vast majority of pediatric fractures, particularly those requiring surgery, were recorded in the KIDS Fracture Tool during the study period^15^.

### Outcomes

The main outcome was the refracture rate at 1 and 2 years in all anatomic regions (excluding the skull and facial bones) between 2014 and 2023. We defined a refracture as a new fracture occurring within 2 years at the same exact location as the primary fracture, confirmed radiographically, while accounting for the effects of bone growth. The secondary outcome was whether the severity of the primary fracture was associated with the refracture risk.

### Inclusion and Exclusion Criteria

**Fig. 1:**
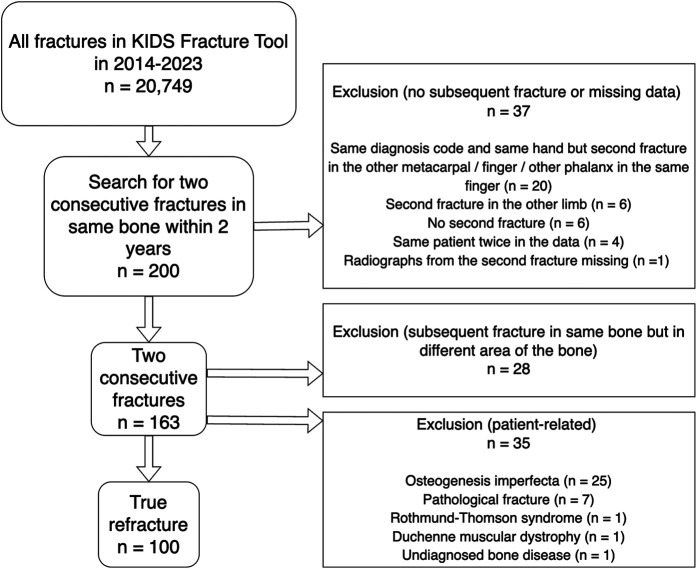
Flowchart of the study population.

All possible refractures that were identified from the KIDS Fracture Tool and occurred within the 2 years after the primary fracture were evaluated. Refractures matching the original fracture site were included in the final analysis. Fractures in other areas of the bone, nonunions, pathological fractures, or refractures in patients with a systemic illness predisposing to multiple fractures (i.e., osteogenesis imperfecta) were excluded (Fig. 1).

### Data Collection

All patients with at least 2 fractures in the same bone between 2014 and 2023 were identified, along with baseline characteristics, from the KIDS Fracture Tool. Patients’ radiographs and patient records were evaluated by the first and last authors. The following data were collected from the radiographs for both the primary fracture and the refracture: fracture location, Peterson classification for epiphyseal fractures, treatment category (A to D from the fracture tool), and any further refractures in the same location. The effect of growth with regard to the fracture location was considered carefully, and cases with any ambiguity were reviewed by all coauthors for consensus. In order to calculate the refracture rate and to compare the distribution of severity (groups A through D) of the primary fracture between patients who did and did not have a refracture within 2 years, the frequency of all fractures in the KIDS Fracture Tool was collected.

This study adhered to the STROBE^16^ (Strengthening of Reporting of Observational Studies in Epidemiology) guidelines.

### Statistical Analysis

Statistical analysis was performed with the use of SPSS (version 28.0; IBM) and R (R Foundation for Statistical Computing). For categorical variables, the frequencies with corresponding percentages were reported. The assumption of normality for continuous variables was assessed by visually inspecting quantile-quantile plots and histograms of the distribution of the values of the variable. The mean and standard deviation were reported for normally distributed variables. The median and range or interquartile range (IQR) were reported for non-normally distributed variables. The difference in the distribution of the fracture treatment types (A through D) was analyzed using the Pearson chi-square test or, when necessary, the Fisher exact test. In addition, relative risks (RRs) of refracture with 95% confidence intervals (CIs) according to fracture severity were calculated. Significance was set at p < 0.05. In this analysis, we compared all fractures combined and the 4 most common anatomic regions with refractures in our data: diaphyseal forearm (International Classification of Diseases, Tenth Revision [ICD-10] codes S52.2, S52.3, S52.4), distal forearm (S52.5, S52.6), distal humerus (S42.4), and diaphyseal tibia (S82.2).

## Results

A total of 20,749 fractures were registered in the KIDS Fracture Tool between 2014 and 2023, and 163 fractures occurred in the same bone within 2 years after the primary fracture. Based on patient records, 35 cases were excluded because of a condition predisposing to fractures. Based on radiographic analysis, 28 cases were excluded because of location mismatch. Finally, 100 confirmed refractures were identified (0.48% refracture rate) (Fig. 1). Of these, 83 occurred within 1 year after the primary fracture and another 17 occurred during the second year.

The mean age (and standard deviation) of the patients at the primary fracture was 9.5 ± 3.3 years (Table I). Most of the patients (69%) were boys. Ninety-one percent of the refractures occurred in an upper extremity, most commonly in the diaphyseal forearm (46%), distal forearm (27%), and distal humerus (11%). In the lower extremities, the diaphyseal tibia was the most common location for a refracture (7%). The median time from the primary fracture to refracture was 110 days (IQR, 68 to 250 days). However, it varied notably between different anatomic regions, from a median of 73 days (IQR, 56 to 131 days) in the distal forearm to a median of 426 days (IQR, 243 to 660 days) in the distal humerus. Additionally, 5 radiographically confirmed second refractures were found. All were in an upper extremity. The vast majority of both primary fractures leading to a refracture and refractures occurred because of a fall or sports-related accident. A total of 16% of the 100 primary fractures leading to a refracture and 37% of the 100 refractures were treated with internal fixation (Table II).

**TABLE I:** Patient Characteristics and Refractures*

Age at primary fracture *(yr)*	9.5 ± 3.3
Sex	
Female	31 (31%)
Male	69 (69%)
Fracture side	
Right	46 (46%)
Left	54 (54%)
Anatomic region of refracture	
Upper limb, total	91 (9%)
Scapula	1 (1.0%)
Clavicula	1 (1.0%)
Proximal humerus	1 (1.0%)
Diaphyseal humerus	1 (1.0%)
Distal humerus	11 (11%)
Diaphyseal forearm	46 (46%)
Distal forearm	27 (27%)
Hand	3 (3.0%)
Lower limb, total	9 (9.0%)
Diaphyseal tibia	7 (7.0%)
Distal fibula	1 (1.0%)
Foot	1 (1.0%)
Time to refracture *(days)*	110 (68 to 250)
Distal humerus	426 (243 to 660)
Diaphyseal forearm	124 (80 to 178)
Distal forearm	73 (56 to 131)
Diaphyseal tibia	109 (79 to 169)
Second refracture	5 (5.0%)
Distal humerus	1 (1.0%)
Diaphyseal forearm	2 (2.0%)
Distal forearm	2 (2.0%)
Third refracture	0 (0.0%)

* The values are given as the mean and the standard deviation; as the number of patients, with the percentage in parentheses; or as the median, with the IQR in parentheses.

**TABLE II:** Fracture Type, Injury Type, and Treatment Method of Refractures and Primary Fractures Leading to a Refracture*

	Primary Fracture	Refracture
Fracture treatment group		
A	15 (15%)	5 (5%)
B	16 (16%)	19 (19%)
C	54 (54%)	43 (43%)
D	15 (15%)	33 (33%)
Torus fracture	5 (5.0%)	2 (2.0%)
Open fracture	2 (2.0%)	1 (1.0%)
Transphyseal fracture	4 (4.0%)	5 (5.0%)
Injury type		
Fall	52 (52%)	53 (53%)
Unorganized sports	26 (26%)	21 (21%)
Organized sports	13 (13%)	19 (19%)
Other	9 (9.0%)	7 (7.0%)
Treatment		
Collar cuff, splint, hard-soled shoe, or no immobilization	10 (10%)	6 (6.0%)
Cast	19 (19%)	18 (18%)
Closed reduction and cast in the emergency department	46 (46%)	28 (28%)
Closed reduction and cast in the operating room	9 (9.0%)	11 (11%)
Operative treatment (flexible intramedullary nail, plate, Kirschner wire)	16 (16%)	37 (37%)

* The values are given as the number of patients, with the percentage in parentheses.

The 2-year refracture rate was clearly highest in both-bone diaphyseal forearm fractures (3.76%), followed by isolated diaphyseal radial (1.41%) and diaphyseal tibial fractures (1.01%) (Table III). The corresponding refracture rate was moderate in the distal humerus (0.58% of supracondylar fractures and 0.49% of all distal humeral fractures combined) and the distal forearm (0.55% [27 of 4,949]). Upper-extremity refractures were uncommon in the proximal forearm (0% [0 of 811]), the isolated diaphysis of the ulna (0% [0 of 136]), the fingers including the thumb (0.04% [1 of 2,697]), and the clavicle (0.11% [1 of 903]). Lower-extremity refractures were uncommon in the femur (0% [0 of 669]), proximal tibia (0% [0 of 474]), distal tibia (0% [0 of 1,095]), and foot (0.05% [1 of 1,883]). No refractures occurred in the spine or pelvis (0% [0 of 316]).

**TABLE III:** Refracture Rate at 1 and 2 Years by Location Based on ICD-10*

Fracture Location (ICD-10 Code)	No. of Primary Fractures	Refractures at 1 Year	Refractures at 2 Years
Clavicle (S42.0)	903	1 (0.11%)	1 (0.11%)
Scapula (S42.1)	13	0 (0.00%)	1 (7.69%)
Proximal humerus (S42.2)	551	1 (0.18%)	1 (0.18%)
Diaphyseal humerus (S42.3)	137	1 (0.73%)	1 (0.73%)
Distal humerus (S42.4)	2,227	4 (0.18%)	11 (0.49%)
Diaphyseal radius (S52.3)	213	3 (1.41%)	3 (1.41%)
Diaphyseal forearm (S52.4)	1,144	40 (3.49%)	43 (3.76%)
Distal radius (S52.5)	3,068	13 (0.42%)	17 (0.55%)
Distal forearm (S52.6)	1,881	10 (0.53%)	10 (0.53%)
Other metacarpal bone (S62.3)	393	2 (0.51%)	2 (0.51%)
Other fingers (S62.6)	2,000	1 (0.05%)	1 (0.05%)
Diaphyseal tibia (S82.2)	693	7 (1.01%)	7 (1.01%)
Lateral malleolus (S82.6)	455	0 (0.00%)	1 (0.22%)
Metatarsal bone (S92.3)	797	0 (0.00%)	1 (0.13%)
All other locations	6,604	0 (0.00%)	0 (0.00%)

* Only anatomic regions with at least 1 refracture are listed individually.

The distribution of the fracture treatment group (A through D) differed in patients who had a refracture compared with patients who did not have a refracture (Table IV). Overall, group C fractures (displaced fracture treated with closed reduction) were associated with an increased risk of refracture compared with group A (RR, 8.0 [95% CI, 4.5 to 14]), group B (RR, 5.0 [95% CI, 2.9 to 8.7]), and group D (RR, 3.2 [95% CI, 1.8 to 5.7]) (Table V). In addition, group D fractures were associated with a higher risk of refracture compared with group A fractures (RR, 2.5 [95% CI, 1.2 to 5.1]). In the diaphyseal forearm, group C was associated with a higher risk of refracture compared with group B (RR, 6.4 [95% CI, 1.6 to 27]). In the distal forearm, both group B fractures (RR, 3.1 [95% CI, 1.0 to 9.5]) and group C fractures (RR, 3.9 [95% CI, 1.4 to 11]) were associated with an increased risk of refracture compared with group A fractures. In the distal humerus, group C was associated with a higher risk of refracture compared with group D fractures (RR, 6.1 [95% CI, 1.1 to 33]). No detectable differences among fracture groups A through D were found in the diaphyseal tibia.

**TABLE IV:** Distribution of Fracture Treatment Groups in Patients Who Did and Did Not Have a True Refracture within 2 Years*

Location	No Refracture				Refracture				P Value
	A	B	C	D	A	B	C	D	
Total	7,438 (40%)	4,930 (26%)	3,289 (18%)	2,975 (16%)	15 (15%)	16 (16%)	54 (54%)	15 (15%)	<0.001
Diaphyseal forearm	86 (7%)	249 (19%)	629 (49%)	313 (25%)	1 (2%)	2 (4%)	34 (74%)	9 (20%)	0.004
Distal forearm	2,085 (43%)	1,062 (22%)	1,362 (28%)	301 (6%)	5 (19%)	8 (30%)	13 (48%)	1 (4%)	0.03
Distal humerus	349 (17%)	517 (25%)	302 (14%)	932 (44%)	4 (36%)	1 (9%)	4 (36%)	2 (18%)	0.02
Diaphyseal tibia	131 (24%)	275 (51%)	65 (12%)	69 (13%)	0 (0%)	3 (43%)	2 (29%)	2 (29%)	0.13

* The values are given as the number of patients, with the percentage in parentheses.

**TABLE V:** Refracture Risk According to Fracture Treatment Group*

Location	Relative Risk Between Fracture Treatment Groups					
	C vs. A	C vs. B	C vs. D	D vs. A	D vs. B	B vs. A
Total	8.0 (4.5 to 14)†	5.0 (2.9 to 8.7)†	3.2 (1.8 to 5.7)†	2.5 (1.2 to 5.1)†	1.6 (0.8 to 3.1)	1.6 (0.8 to 3.2)
Diaphyseal forearm	4.5 (0.6 to 32)	6.4 (1.6 to 27)†	1.8 (0.9 to 3.8)	2.4 (0.3 to 19)	3.5 (0.8 to 16)	0.7 (0.1 to 7.6)
Distal forearm	3.9 (1.4 to 11)†	1.2 (0.5 to 3.0)	2.9 (0.4 to 22)	1.4 (0.2 to 12)	0.4 (0.1 to 3.5)	3.1 (1.0 to 9.5)†
Distal humerus	1.1 (0.3 to 4.5)	6.8 (0.8 to 60)	6.1 (1.1 to 33)†	0.2 (0.03 to 1.0)	1.1 (0.1 to 12)	0.2 (0.02 to 1.5)
Diaphyseal tibia	9.7 (0.5 to 199)	2.7 (0.5 to 16)	1.1 (0.2 to 7.3)	9.1 (0.4 to 188)	2.6 (0.4 to 15)	3.3 (0.2 to 64)

* The values are given as the RR, with the 95% CI in parentheses.

† Significant.

## Discussion

We found the 2-year refracture rate among individuals <16 years of age in the primary catchment area to be 0.48%. Most refractures occurred within the first year, with 5% experiencing a second refracture later in the same bone. Refracture rates were highest in the diaphyseal both-bone forearm fractures, followed by the diaphyseal tibia, the distal humerus, and the distal forearm. The median time to refracture varied by anatomic region, with the shortest time in the distal forearm and the longest time in the distal humerus. Displaced primary fractures treated with closed reduction showed an increased refracture risk compared with nondisplaced fractures and fractures treated with internal fixation.

Our study found a 2-year consecutive fracture rate of 0.79%, comparable with the overall refracture rate reported in the Swedish Fracture Register (0.88% within 1 year)^14^. However, upon excluding patients with consecutive fractures representing unrelated incidents rather than true refractures and patients with conditions predisposing them to multiple fractures, notably osteogenesis imperfecta, our data revealed a significantly lower refracture rate (0.40% at 1 year and 0.48% at 2 years). A historical cohort study from Sweden in 1975 to 1979 reported a 0.5% (21 of 4,621) refracture rate^17^. To our knowledge, no other studies have assessed the overall refracture rate in children.

Forearm refractures represented 73% of all refractures in our data. In the diaphyseal forearm the refracture rate in both-bone fractures was notable at 3.8%. This is in line with previous studies. Amilon et al. reported a 3.4% refracture rate within 1 year for diaphyseal forearm fractures in the Swedish Fracture Register^14^. Bould and Bannister reported a 4.9% refracture rate for displaced forearm fractures^10^, whereas Tisosky et al. reported a clearly lower refracture rate of 1.9% in 2,590 patients^12^.

The median time to refracture was similar in the diaphyseal forearm in our data compared with the Swedish report^14^. However, our median time to refracture in the distal forearm was shorter (73 days compared with 180 days), and the refracture rate was slightly lower (0.55% compared with 0.68%) compared with the Swedish report. Notably, in our study, 71% (20) of the 28 fractures that were excluded because they were found to be located in a different part of the bone than the original fracture were distal forearm fractures. Additionally, the distal forearm is the most common fracture location in children^18^, and represented 24% of all fractures in our data set (Table III). Therefore, it is likely that the distal forearm refractures reported by Amilon et al. include some proportion of new fractures unrelated to the primary fractures^14^.

The overall median time to refracture was lower in our study (110 days) compared with the findings from the Swedish Fracture Register (140 days), even though our follow-up included 2 years instead of 1 year. In contrast, in the distal humerus, the median time to refracture was 426 days in our study whereas it was only 192 days in the Swedish Fracture Register, and 7 of the 11 distal humeral refractures in our data occurred after 1 year. With regard to the diaphyseal tibia, both the refracture rate and the median time to refracture were comparable between our findings and the results from the Swedish Fracture Register.

The association between the severity of the primary fracture and the risk of refracture has not been assessed before. Based on our findings, displaced fractures treated with closed reduction (group C) were associated with the highest risk of refracture. In the diaphyseal forearm, where the overall refracture rate was highest, the risk of refracture was >6 times higher in group C fractures compared with fractures requiring follow-up but not reduction (group B) and tended to be higher compared with fractures treated using internal fixation (group D). Interestingly, in the distal humerus, group C fractures were associated with >6 times higher risk of refracture compared with group D fractures. It could be hypothesized that malposition of the fracture may predispose to refracture, as fractures treated with internal fixation are typically better reduced compared with fractures treated with closed reduction. Internal fixation may also protect the injured bone from further fractures after the immobilization period ends, whereas higher-energy trauma in the primary fracture may prolong healing. Therefore, the fracture treatment type should be taken into account when considering the recommended period of caution after the immobilization to minimize the risk of refracture, especially in nonoperatively treated fractures.

Our study had both strengths and limitations. With regard to strengths, first, this analysis was based on >20,000 fractures treated at our institution. Second, our setting enabled a detailed analysis of patient records and radiographs, and we were thus able to distinguish between true refractures and unrelated subsequent fractures in the same bone. Third, we excluded patients with conditions predisposing to refractures. Fourth, we were able to find novel information on the relationship between fracture treatment type and risk for refracture.

With regard to limitations, first, despite a large number of primary fractures, the number of refractures remained limited. Therefore, we also included refractures that occurred in 2023 in order to achieve the maximal sample size, even though the follow-up was <2 years in fractures that occurred after 2022. This may have slightly underestimated the refracture rate; however, the effect was likely marginal because a majority of the refractures occurred within a few months after the primary fracture. There was a possibility that some refractures were treated elsewhere, which would have underestimated the rate of refractures in our study; however, we doubt that this would have affected our results substantially. Second, our method of dividing fractures to A through D types by treatment was not absolute, as an overlap in treatment methods, especially regarding the forearm, exists^19-21^. The rough grouping of fractures in the KIDS Fracture Tool was a notable limitation of the study, as it did not classify fractures by recognized pediatric fracture types and instead focused on the treatment received. However, it did indicate that refractures were more common in displaced fractures, which are associated with higher-energy injury.

In conclusion, refractures were found to be uncommon in children, with an overall rate of approximately 0.5%. The highest refracture rates were observed in the both-bone diaphyseal forearm fractures, followed by the tibia, the distal humerus, and the distal forearm. The median time to refracture varied across anatomic regions. Displaced fractures treated with closed reduction were associated with an increased risk of refracture.

## Appendix

Supporting material provided by the authors is posted with the online version of this article as a data supplement at jbjs.org (http://links.lww.com/JBJS/I521).
